# The Diverse Activities and Mechanisms of the Acylphloroglucinol Antibiotic Rhodomyrtone: Antibacterial Activity and Beyond

**DOI:** 10.3390/antibiotics13100936

**Published:** 2024-10-02

**Authors:** Rupa Rani, Gabriela Marinho Righetto, Ann-Britt Schäfer, Michaela Wenzel

**Affiliations:** 1 Division of Chemical Biology, Department of Life Sciences, Chalmers University of Technology, 412 96 Gothenburg, Sweden; 2 Centre for Antibiotic Resistance Research in Gothenburg (CARe), 413 45 Gothenburg, Sweden

**Keywords:** rhodomyrtone, *Rhodomyrtus tomentosa*, acylphloroglucinol, plant-derived antimicrobials, natural product, mode of action, antimicrobial activity

## Abstract

**Background/Objectives:** The rose myrtle *Rhodomyrtus tomentosa* is a medicinal plant used in traditional Asian medicine. The active compound in *R. tomentosa* leaf extracts is rhodomyrtone, a chiral acylphloroglucinol. Rhodomyrtone exhibits an impressive breadth of activities, including antibacterial, antiviral, antiplasmodial, immunomodulatory, and anticancer properties. Its antibacterial properties have been extensively studied. **Methods:** We performed a comprehensive literature review on rhodomyrtone and summarized the current knowledge about this promising acylphloroglucinol antibiotic and its diverse functions in this review. **Results:** Rhodomyrtone shows nano to micromolar activities against a broad range of Gram-positive pathogens, including multidrug-resistant clinical isolates, and possesses a unique mechanism of action. It increases membrane fluidity and creates hyperfluid domains that attract membrane proteins prior to forming large membrane vesicles, effectively acting as a membrane protein trap. This mechanism affects a multitude of cellular processes, including cell division and cell wall synthesis. Additionally, rhodomyrtone reduces the expression of inflammatory cytokines, such as TNF-α, IL-17A, IL1β, and IL8. Generally showing low toxicity against mammalian cells, rhodomyrtone does inhibit the proliferation of cancer cell lines, such as epidermal carcinoma cells. The primary mechanism behind this activity appears to be the downregulation of adhesion kinases and growth factors. Furthermore, rhodomyrtone has shown antioxidant activity and displays cognitive effects, such as decreasing depressive symptoms in mice. **Conclusions:** Rhodomyrtone shows great promise as therapeutic agent, mostly for antibacterial but also for diverse other applications. Yet, bottlenecks such as resistance development and a better understanding of mammalian cell toxictiy demand careful assessment.

## 1. Introduction

The indiscriminate and extensive use of antibiotics has led to the emergence of antimicrobial resistance (AMR), resulting in a considerable decrease in effective treatment options and, consequently, severe complications and increased numbers of AMR-associated deaths [1,2]. The World Health Organization (WHO) considers AMR one of the major global health risks [3] and has published lists of bacterial and fungal pathogens for which new treatments are most urgently needed [4,5], including resistant strains of *Staphylococcus aureus*, *Streptococcus pneumoniae*, and *Enterococcus faecalis/faecium*. New antimicrobial drugs able to combat these resistant pathogens are urgently needed. However, despite multilateral efforts to promote new antibacterial drug development, the clinical pipeline is still limited, particularly with respect to innovative drugs with activity against critical pathogens. Thus, the discovery void that has plagued antimicrobial development since the 1980s still persists [6].

Natural compounds produced by plants seem to be an interesting and promising source of new pharmaceuticals and have become an emerging category of therapeutic agents [7]. Limited data are available on the mechanisms underlying antimicrobial compounds from the plant kingdom, and currently, no plant-derived antibiotic is approved for clinical use. This is surprising given the plethora of natural, plant-based remedies that are used as anti-infectives in traditional medicine by cultures from all continents [8]. To unlock the potential of this traditional knowledge for modern medicine, research into the activities and mechanisms of plant-derived pharmaceuticals is pivotal.

**Figure 1:**
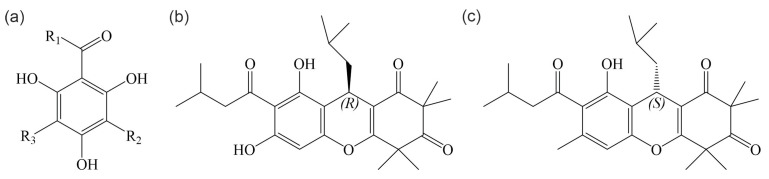
Structures of acylphloroglucinols. (**a**) General structure of acylphloroglucinol compounds. (**b**) Structure of (R)-rhodomyrtone. (**c**) Structure of (S)-rhodomyrtone.

The rose myrtle *Rhodomyrtus tomentosa* (Aiton) Hassk. has many traditional uses in Asian culture, including food (e.g., wine and jam) and medical applications. Particularly in China, Malaysia, Vietnam, Thailand, and Indonesia, various parts of the plant (roots, trunk, crushed leaves, fruits, and flowers) are used to treat a number of ailments. For example, there are records of the plant being used to treat diarrhea, dysentery, fever, pain, heartburn, and even snake bites [9,10,11,12]. Importantly, *R. tomentosa* leaf and root concoctions are commonly used to treat oral, gastrointestinal, and urinary tract infections, as well as infections associated with childbirth. Concoctions are further used as antiseptic wound washes, and crushed leaves are applied as wound dressings to prevent infection after injury [13,14,15]. A plethora of scientific studies has confirmed the antimicrobial potency of *R. tomentosa* leaf extracts, mainly attributed to the acylphloroglucinol rhodomyrtone (Figure 1).

In this review, we cover the antimicrobial activity of this unusual antibacterial compound, including its spectrum of activity, mechanism of action, antibiofilm potential, and resistance mechanisms. Further, we summarize other medically relevant bioactivities and mechanisms of rhodomyrtone, such as immunomodulatory and anticancer activity, as well as possible concerns, such as toxicity potential and antimicrobial resistance. To the best of our knowledge, this is the first comprehensive literature review of the diverse bioactivities and mechanisms of rhodomyrtone.

## 2. Chemical Description of Rhodomyrtone

Acylphloroglucinol compounds (Figure 1a) are derivatives of phloroglucinol (1,3,5-trihydroxy benzene), a major class of secondary metabolites. Acylphloroglucinols are considered the largest group of compounds among phloroglucinols of natural origin. They can be composed of one or more rings and are characterized by the presence of a CRO group [16]. Acylphloroglucinols have shown broad biological activities, including antibacterial, antimalarial, anticancer, and antioxidant activities, leading to considerable efforts to synthesize these compounds [17]. Rhodomyrtone is a natural acylphloroglucinol compound that occurs in the rose myrtle *R. tomentosa* and is typically isolated from the leaves of the plant by ethanol extraction followed by purification by medium-pressure liquid chromatography [18]. Natural extracts contain a mixture of (R)- and (S)-rhodomyrtone isomers (Figure 1b,c). Differences in the biological activity of the two enantiomers have not yet been explored. Therefore, here we refer to the racemic mix as ‘rhodomyrtone’. Unless specified otherwise, the studies reviewed here have been performed with a mix of both enantiomers. Rhodomyrtone is an uncharged molecule with several polar and nonpolar groups that are evenly distributed over its structure. This distinct lack of amphipathicity sets rhodomyrtone apart from other antimicrobial molecules that share the same molecular target, the bacterial cell membrane, as membrane-active antibacterial compounds are usually positively charged and/or amphipathic [19].

## 3. Antimicrobial Activity

Many studies have demonstrated that rhodomyrtone is active against a broad range of Gram-positive bacteria, such as *S. aureus,* including high-risk MRSA strains, *Staphylococcus epidermidis*, *S. pneumoniae*, *Streptococcus pyogenes*, and *Streptococcus mutans*. Minimal inhibitory concentrations (MICs) and minimal bactericidal concentrations (MBCs) against Gram-positive bacteria are typically in the nano to low microgram per milliliter range and, thus, competitive with clinically used antibiotics. In most cases, the MIC did not significantly increase in multidrug-resistant strains compared to susceptible strains. With the exception of vancomycin-resistant *enterococci* (VRE), MBCs were rarely considerably higher than the MIC against the corresponding strain (Table 1).

One of the many applications suggested for rhodomyrtone is the prophylaxis and treatment of inflammatory acne lesions. Thus, the activity of both *R. tomentosa* leaf extracts and purified rhodomyrtone against *Propionibacterium acnes*—a Gram-positive bacterial skin commensal commonly associated with acne—has been thoroughly tested, typically presenting MICs between 0.125 and 2 µg/mL [20,21,22,23]. In addition to good in vitro activity, rhodomyrtone also significantly reduced inflammatory acne lesions in human volunteers [23].

Rhodomyrtone does not show activity against Gram-negative bacteria [24]. However, the compound does interact with liposomes made from *E. coli* polar lipid extracts in a manner similar to its interaction with the cell membrane in Gram-positive cells [25], suggesting that its inactivity is not due to an inability to interact with its target but rather an inability to pass the impermeable outer membrane of Gram-negative bacteria.

Rhodomyrtone also did not show activity against yeast [24,26]. While the factors underlying the specificity and selectivity of rhodomyrtone have yet to be systematically explored, it did not affect the membranes of human erythrocytes and exhibited a preference for phosphatidylglycerol (PG) and phosphatidylethanolamine (PE) over phosphatidylcholine (PC) [25], suggesting that it prefers prokaryotic cell membranes. It should, however, be noted that *R. tomentosa* crude leaf extracts inhibited the growth of *Candida albicans* at very high concentrations (1 mg/mL) and led to a clear, concentration-dependent reduction of *C. albicans* adhesion to both plastic surfaces and human buccal cells. Interestingly, purified rhodomyrtone, despite not showing growth inhibition up to the highest tested concentration of 100 µg/mL, clearly impaired *C. albicans* adhesion as well [26], suggesting that antifungal applications of rhodomyrtone are not entirely off the table.

In addition to its antibacterial and limited antifungal effects, rhodomyrtone also showed moderate antiplasmodial activities against the chloroquine-sensitive 3D7 strain of *Plasmodium falciparum* (1.84 mM) and the chloroquine-resistant Dd2 strain (4 mM) [27].

**Table 1:** Antimicrobial activity of rhodomyrtone.

Species	Strain	MIC (µg/mL)	MBC (µg/mL)	References
**Gram-positive bacteria**				
*Staphylococcus aureus*	ATCC 29213	0.5–2	1–16	[28,29,30,31,32]
	ATCC 25923	0.25–0.78	0.39–1	[18,21,26]
	ATCC 6538	1.83	n.d.	[33]
	NRIC 1135	0.78	n.d.	[24]
	RN4220 (ATCC 35556)	0.5	n.d.	[34]
	Newman	0.5	0.625	[35]
	COL	0.5	1	
	75 clinical isolates	0.25–2	0.5–8	
	110 clinical MRSA isolates	1	4–8	[36]
	ATCC 29740 (bovine MSSA)	0.5	0.5	[37]
	1158c (bovine MRSA)	0.5	1	
	4 mastitis isolates	0.5	1	
	NPRC 302	1	2	[21]
	NPRC 308	0.5	1	
	NPRC 317	0.25	1	
	NPRC 322	1	2	
	NPRC R001 (MRSA)	0.5–1	1–4	[28,29,38]
	EMRSA-16	0.5–1	0.5–4	[30,32,39]
	EMRSA-15	1	1	[30]
	EMRSA SOTON9	0.5	1	
	MRSA BB270	0.5	0.5	
	MRSA USA300	1	1	
	VISA Mu3	0.5	1	
	VISA Mu50	0.5	0.5	
	4 MRSA isolates	0.39–0.78	0.39–0.78	[18]
*Staphylococcus epidermidis*	ATCC 35984	0.25–8	2–25	[18,21,31]
	NBRC 100911	0.78	n.d.	[24]
	NPRC 529	0.5	1	[21]
	NPRC 537	0.5	2	
	NPRC 573	0.25	2	
	NPRC 577	0.25	1	
*Staphylococcus simulans*	3100-0949	0.5	1	[37]
*Staphylococcus chromogenes*	3140-3115	0.25	1	[37]
Coagulase-positive staphylococci	BMPOS-31 (mastitis isolate)	4	32	[31]
Coagulase-negative staphylococci	BMNEG-12 (mastitis isolate)	2	16	[31]
*Streptococcus pneumoniae*	ATCC 700673	0.5	1	[40]
	R6	2	8	
	TIGR4	1	2	
	23 clinical isolates	0.125–4	0.125–8	
	not specified	0.39	1.56	[18]
*Streptococcus pyogenes*	2 clinical isolates	0.39–0.78	1.56	[18]
	47 clinical isolates	0.39–1.56	0.39–1.56	[13]
*Streptococcus mutans*	JCM 5175	1.56	n.d.	[24]
	clinical isolate	0.39	n.d.	[26]
	not specified	0.19	1.56	[18]
*Streptococcus suis*	P1/7	0.5	1	[41]
*Streptococcus gordonii*	not specified	0.19	1.56	[18]
*Streptococcus salivarius*	not specified	0.39	1.56	[18]
*Enterococcus faecalis*	ATCC 29212	2	32	[30]
	not specified	1.56	12.5	[18]
*Enterococcus* spp.	VRE-2	2	>32	[30]
	VRE-3	1	16	
	VRE-4	1	32	
	VRE-7	2	>32	
	VRE-8	2	>32	
*Propionibacterium acnes*	DMST 14916	0.25	0.25	[21]
	NPRC 021	0.25	0.5	
	NPRC 036	0.25	0.25	
	NPRC 039	0.25	0.25	
	9 clinical isolates	0.125–0.5	0.25–0.5	[22]
*Clostridium difficile*	10 clinical isolates	0.625–2.5	1.25–5	[42]
*Bacillus cereus*	NBRC 3457	0.78	n.d.	[24]
	ATCC 11778	0.5	4	[43]
	65 food isolates	0.5	2–8	
	not specified	0.39	0.78	[18]
*Bacillus subtilis*	168CA	0.5	n.d.	[25,44]
	JCM 1465	0.78	n.d.	[24]
	not specified	0.39	0.39	[18]
*Micrococcus luteus*	NBRC 12708	0.78	n.d.	[24]
**Gram-negative bacteria**				
*Escherichia coli*	O157 JCM 18426	>100	n.d.	[24]
*Pseudomonas aeruginosa*	JCM 5962	>100	n.d.	[24]
*Salmonella typhimurium*	NBRC 12529	>100	n.d.	[24]
**Fungi**				
*Candida albicans*	ATCC 90028	>100	n.d.	[26]
	JCM 2085	>100	n.d.	[24]
*Saccharomyces cerevisiae*	NRIC 1410	>100	n.d.	[24]

MIC: minimal inhibitory concentration, MBC: minimal bactericidal concentration, MSSA: methicillin-sensitive *S. aureus*, MRSA: methicillin-resistant *S. aureus*, EMRSA: epidemic methicillin-resistant *S. aureus*, VISA: vancomycin-intermediate *S. aureus.* n.d.: not determined.

**Figure 2:**
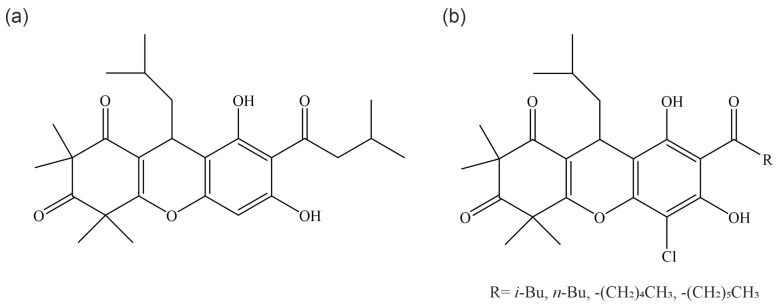
Structures of rhodomyrtone (**a**) and C7-modified rhodomyrtone derivatives with improved activity against *S. aureus*.

Chemical modification of isolated rhodomyrtone is possible but has not led to improvements in activity [45]. The total synthesis of rhodomyrtone, as well as its naturally occurring isomer rhodomyrtosone B, has also been reported [46,47]. Synthesized rhodomyrtone and rhodomyrtosone B have shown comparable activity to their isolated counterparts against *S. aureus* [46,48,49]. A series of rhodomyrtone derivatives have been synthesized, and some new compounds showed 2–4-fold increased activity against *S. aureus* (Figure 2). No further characterization of these compounds has yet been performed to assess, e.g., toxicity and mode of action, and further studies will be needed to evaluate whether the new derivatives are superior to rhodomyrtone in other aspects than activity against *S. aureus* [50].

## 4. Antibiofilm Activity

Rhodomyrtone has also been implicated in the inhibition of biofilm formation and dispersal of mature biofilms. Biofilms are complex and structured bacterial communities, physiologically different from planktonic organisms. This microbial community grows in a surface-associated manner, embedded in an extracellular matrix that protects the bacteria from hostile environments, including immune factors and antibiotics. Biofilm formation is an important pathogenesis parameter of many bacterial infections [51]. Thus, antibiofilm activity is a very desirable characteristic in an antimicrobial compound.

Wunnoo et al. found that rhodomyrtone was not only able to inhibit both lipase production and biofilm formation but also eradicated bacteria within mature *P. acnes* biofilms [22]. Thereby, 1/16 to 1/8 of the MIC were sufficient to achieve a significant (*p* < 0.05) reduction in the biofilm formation of clinical isolates. *P. acnes* viability within mature biofilms upon treatment with 4–8 × the MIC ranged between 40% and 85% [22]. *P. acnes* forms biofilms within the follicle, making acne treatment with non-biofilm-penetrating drugs inefficient. The antibiofilm activities of rhodomyrtone thus relate well to its favorable acne-treating properties [21,23,52].

Rhodomyrtone has also shown promise in antibiofilm activity against oral pathogens, which constitute one of the most common health problems associated with biofilm formation. Thus, rhodomyrtone prevented the adhesion of *S. aureus*, *S. mutans*, and *C. albicans* to both plastic surfaces and human buccal cells [26]. Adhesion is the initial and essential step of biofilm formation. Biofilm-forming dental pathogens cause dental plaques that are responsible for acute and chronic infections like caries and periodontitis and pose a particular concern for people wearing braces, dentures, and implants [53]. Interestingly, rhodomyrtone possesses further activities that can help combat dental disease. Bach et al. showed that rhodomyrtone reduces the biofilm formation of *S. mutans* by up to 59% and has bactericidal activity against cells within biofilms. Importantly, they also found that it inhibited the bacterial enzymes for acid production and tolerance, namely F-ATPase, the phosphotransferase system, glyceraldehyde-3-phosphate dehydrogenase, and pyruvate kinase [54]. Acid production is a major factor in the pathogenesis of caries caused by this bacterium. Thus, rhodomyrtone counteracts oral biofilm and disease on multiple levels, including adhesion, biofilm formation, dispersion of mature biofilms, killing of cells within biofilms, and inhibition of cariogenic metabolic pathways in oral pathogens.

Further studies have shown the antibiofilm activity of rhodomyrtone against *staphylococci* and *streptococci*. Thus, the compound is able to reduce biofilm formation and eradicate mature biofilms in a dose-dependent manner in *S. aureus* and *S. epidermidis* [52]. An ex vivo study in a bovine udder epidermal tissue model showed that both pure rhodomyrtone and liposomal-encapsulated rhodomyrtone decreased *S. aureus* adhesion to the tissue [31]. Rhodomyrtone was able to prevent biofilm formation of *S. pneumoniae*, the leading cause of pneumonia and meningitis in adults, at 1/8 of the MIC in different clinical isolates [55]. In *S. pyogenes*, the inhibition of biofilm formation by rhodomyrtone was pinpointed to quorum sensing inhibition [56].

## 5. Antibacterial Mechanism of Action

The identification of an antibacterial mechanism of action can be a challenging task, especially when the antibiotic in question possesses an entirely new mechanism of action. This challenge is reflected in the history of rhodomyrtone, for which a number of different mechanisms have been proposed based on a plethora of experiments in diverse models. For a long time after its discovery, seemingly contradicting findings and hypotheses have caused confusion about the antibacterial mechanism of this antibiotic. For example, studies conducted on *C. difficile* and MRSA showed bacteriolytic effects [38,42], while others on *S. pyogenes* and *B. subtilis* reported no bacteriolytic activity and suggested that the primary mechanism of bactericidal action is not related to membrane pores or large-scale cell lysis [25,56,57,58]. The molecular mass of rhodomyrtone is low (442.6 g/mol) [59], and uptake studies have found the compound in cytosolic cell fractions [30], leading to the belief that rhodomyrtone can penetrate bacterial cells and inhibit intracellular targets. Molecular modeling studies have put forward dihydrofolate reductase (DHFR) as a target, but that idea was immediately disproven by checkerboard assays with the DHFR inhibitor trimethoprim [34]. Instead, the major cell division protein FtsZ was proposed as a target, supported by both molecular modeling and transcriptomic profiling [29,34,39]. Indeed, cell division defects were observed in *S. aureus*, *B. subtilis*, and *S. suis* cells [34,41,44] but ultimately proven to be a consequence of secondary effects on cell division proteins [25,44]. Finally, studies on its membrane activity could provide evidence for a new mechanism of action affecting a range of pathways [25,58], paving the way for a new mode of action model that could explain and unify the seemingly conflicting observations of earlier studies. In the following, we will give an overview of mechanistic studies on rhodomyrtone, the current mode of action model, and how it sets rhodomyrtone apart from other membrane-active compounds.

### 5.1. Effects on Cell Division and FtsZ

FtsZ (filamenting temperature-sensitive) is a bacterial actin homolog that drives cell division by forming the constricting Z-ring [60]. FtsZ possesses a GTPase domain that allows it to hydrolyze GTP to GDP and phosphate (GTPase activity), driving polymerization into FtsZ filaments that assemble into the Z-ring at mid-cell. The Z-ring then constricts and, driving septation, separates the cell into two daughter cells [61]. Subsequently, FtsZ disassembles, and GDP is released from FtsZ, which is then ready to bind a new GTP molecule and polymerize again [62,63]. The Z-ring is a dynamic structure that is constantly polymerizing on one end and de-polymerizing on the other end, which is known as treadmilling and is essential for Z-ring constriction and septum formation [64,65]. This process is vital for bacterial cytokinesis, and the FtsZ protein is essential for almost all bacteria.

Proteome and transcriptome profiling of *S. aureus* treated with rhodomyrtone revealed a down-regulation of FtsZ [29,39], leading to the hypothesis that it could be the molecular target of rhodomyrtone. Molecular docking studies suggested that the compound could indeed bind to the nucleotide-binding pocket of the FtsZ protein, whereby the (S)-enantiomer bound more strongly than the (R)-enantiomer. This finding was initially supported by an enlarged cell phenotype in phase contrast microscopy of rhodomyrtone-treated *S. aureus* [34].

Due to the better affinity of rhodomyrtone than GDP and GTP in the molecular dynamics simulations, it was speculated that (S)-rhodomyrtone, binding to the nucleotide-binding site of FtsZ, would compete with GDP in monomeric FtsZ, thereby obstructing the ‘reloading’ of FtsZ with GTP and thus resulting in inhibition of FtsZ polymerization. Moreover, conformational changes in the GTPase domain observed upon binding of (S)-rhodomyrtone could further impair the function of FtsZ [44]. Indeed, predictions made based on these simulations could be confirmed in in vitro experiments with purified *B. subtilis* FtsZ. Thus, in the presence of rhodomyrtone, the GTPase activity was decreased concentration-dependently by up to 45%, and FtsZ polymerization was reduced by 36% [44]. However, rhodomyrtone did not cause any protein aggregation or aberrant FtsZ bundle formation, as observed with other FtsZ inhibitors [66]. Despite the consistency of in silico and in vitro data, FtsZ could ultimately not be confirmed as a primary target of rhodomyrtone in vivo. While phase contrast microscopy showed cell swelling and deformation [44], the typical elongation observed upon FtsZ inhibition in *B. subtilis* [67] was not observed, suggesting inhibition of cell elongation rather than division. Further, the localization of FtsZ (Z-ring formation) was not displaced by rhodomyrtone until 60 min of treatment, suggesting a downstream effect on this protein [44]. It was suggested that this late displacement might be a consequence of membrane depolarization since the membrane anchors of FtsZ, FtsA, and SepF depend on the transmembrane potential and lose their membrane binding upon depolarization, consequently leading to the displacement of FtsZ [68]. Indeed, both membrane anchors were displaced after rhodomyrtone treatment, suggesting a membrane-related mechanism of action rather than a specific inhibition of FtsZ [44].

Despite most likely being an indirect downstream effect, the inhibition of cell division by rhodomyrtone is consistent with further phenotypic observations, including early observations of cell division defects in *S. aureus* [29]. More recently, Traithan et al. have observed clear cell division aberrations in *S. suis*, including incomplete nucleoid segregation and septum misplacement, either over unsegregated nucleoids or close to cell poles, resulting in multiple constriction sites and/or anucleated cells [41]. Importantly, this phenotype originated from the accumulation of FtsZ in fluid membrane domains, leading to its misplaced activity. This finding tied together observations of cell division defects and the new mode of action model (described below), which is based on the generation of fluid membrane domains that attract and trap membrane proteins [25].

### 5.2. Interaction with Bacterial Membranes

Following the observation that FtsZ, FtsA, and SepF all delocalized after rhodomyrtone treatment, the focus of the mode of action elucidation shifted from intracellular targets to the cell envelope, especially the cell membrane. Originally, Leejae et al. reported that rhodomyrtone accumulated in the cytoplasmic fraction [30]. However, this experiment was conducted after 18 h of incubation, a time point at which most cells have already died [37]. A more recent experiment examined shorter time points between one and four hours and found an accumulation of rhodomyrtone in the membrane and cell wall fractions [32], consistent with the notion that the target must be in the cell envelope.

Radioactive precursor incorporation experiments revealed a concentration-dependent inhibition of all tested pathways, comprising DNA, RNA, protein, cell wall, and lipid synthesis [32]. This behavior is typical for antibiotics that target the cell membrane as energy limitation due to membrane depolarization and respiratory chain inhibition, leading to a shutdown of all energy-dependent cellular reactions. When localization reporters for intracellular processes (DNA, RNA, protein synthesis) were tested with rhodomyrtone, no effects were observed [25], corroborating the idea of indirect effects due to energy depletion. When membrane-bound reporters were used, however, all tested proteins showed accumulation in large membrane-associated clusters, the only exception being FtsA, which, as observed before [44], lost its membrane binding entirely [25], likely due to its high sensitivity to membrane depolarization [68].

**Figure 3:**
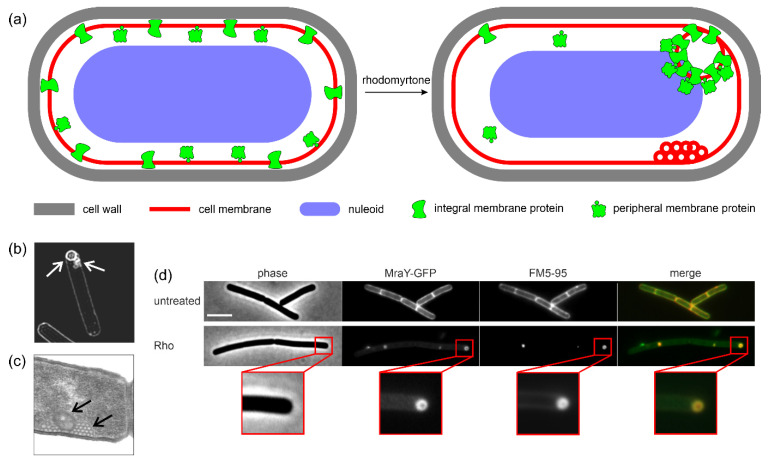
Effects of rhodomyrtone on bacterial cells. (**a**) Schematic overview of cellular effects. (**b**) Rhodomyrtone-induced vesicles visualized with structured illumination microscopy. (**c**) Vesicles visualized with transmission electron microscopy. Membranes were stained with Mitotracker green. (**d**) Trapping of the integral membrane protein MraY in rhodomyrtone-induced vesicles. Membranes were stained with FM5-95. Panels (**b**–**d**) show *Bacillus subtilis* cells and were reproduced from [25].

Indeed, the depolarization and impairment of cellular respiration could be confirmed using reporter dyes. While pore formation could be excluded, slow potassium leakage was observed, correlating with depolarization [25]. However, depolarization did not explain the accumulation of membrane proteins in large clusters. An explanation for this phenotype could be provided by a combination of fluidity-sensitive membrane dyes, timelapse, super-resolution, and electron microscopy (Figure 3). Thus, rhodomyrtone fuses naturally occurring fluid membrane microdomains (coined regions of increased fluidity, RIFs [69]) into large, hyperfluid domains that attract diverse membrane proteins, including both peripheral and integral membrane proteins, fulfilling a range of cellular functions. In the second step, promoted by the high local membrane fluidity and the inherent ability of rhodomyrtone to induce membrane curvature, the membrane bends and forms large vesicles that irreversibly trap the accumulated membrane proteins (Figure 3). Large protein-trapping vesicles of 50–100 nm diameter, but also smaller (~10 nm) vesicles that accumulated in clusters and did not trap detectable amounts of protein, were formed (Figure 3b,c). This mechanism has been described as a membrane protein nanotrap and has been observed in *B. subtilis*, *S. aureus*, and *S. pneumoniae* [25]. Similar observations were later made for *S. suis* as well [41].

Further insight into the membrane interaction of rhodomyrtone was provided by in silico and in vitro experiments. Molecular dynamics simulations indicated that rhodomyrtone transiently binds to both PG and PE head groups but does not intercalate between membrane lipids. This binding causes a ‘pulling’ effect, resulting in increased membrane disorder, i.e., fluidity and curvature. Indeed, both the affinity for PG and PE and the ability to bend membranes have subsequently been confirmed in vitro using liposomes [25].

The apparently unselective trapping and delocalization of membrane proteins that affect a variety of cellular functions matches the results of transcriptomic and proteomic studies, which have shown differentially regulated genes/proteins belonging to a range of processes including, for example, lipid metabolism, amino acids, and nucleic acid synthesis [29,39,40,58].

### 5.3. Comparison with Other Non-Pore-Forming, Membrane-Active Antimicrobials

**Figure 4:**
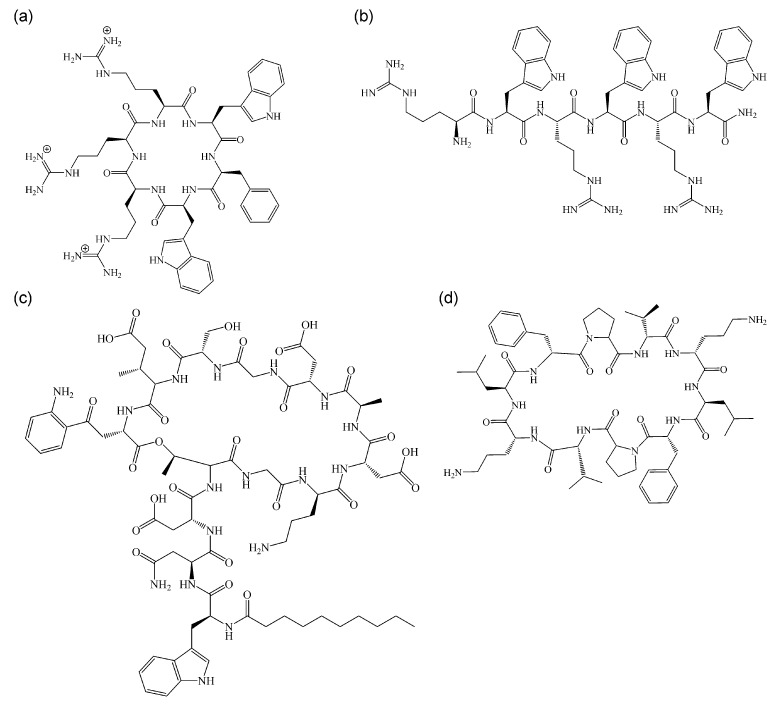
Structures of non-pore-forming membrane-active antimicrobials with well-characterized mechanisms. (**a**) cWFW (cycloRRRWFW), (**b**) MP196 (RWRWRW-NH_2_), (**c**) daptomycin (N-Decanoyl-Trp–D-Asn–Asp–Thr–Gly–Orn–Asp–D-Ala–Asp–Gly–D-Ser–3-Me-Glu–Kyn), and (**d**) gramicidin S (cyclo(-Val-Orn-Leu-D-Phe-Pro-)_2_).

The protein-trapping mechanism of rhodomyrtone is unique, and no other compound has shown comparable activities. Yet, rhodomyrtone can be classed as a non-pore-forming membrane-active antimicrobial, a mechanistic class consisting of mainly antimicrobial peptides (AMPs) and other peptide-based antibiotics. Membrane activity has been explained by plain pore formation for decades, yet this model has been challenged following the availability of sensitive methods to study antibiotic-membrane interaction in living bacterial cells. Thus, some mode of action models of ‘established’ pore formers had to be revised, and several more compounds were discovered that impair bacterial membranes without inducing pores [70]. Not many non-pore-forming membrane-active antimicrobials have been studied in as great mechanistic detail as rhodomyrtone, but from those that are well-characterized, we can conclude that membrane fluidity and membrane protein localization are two key factors in their activity [25,71,72,73,74]. In the following, we compare the mechanism of action of rhodomyrtone with those of the lipopeptide daptomycin, the short cationic AMPs cWFW and MP196, and the cyclic β-sheet peptide gramicidin S (Figure 4), which have been thoroughly characterized with a similar methodology to rhodomyrtone.

**Figure 5:**
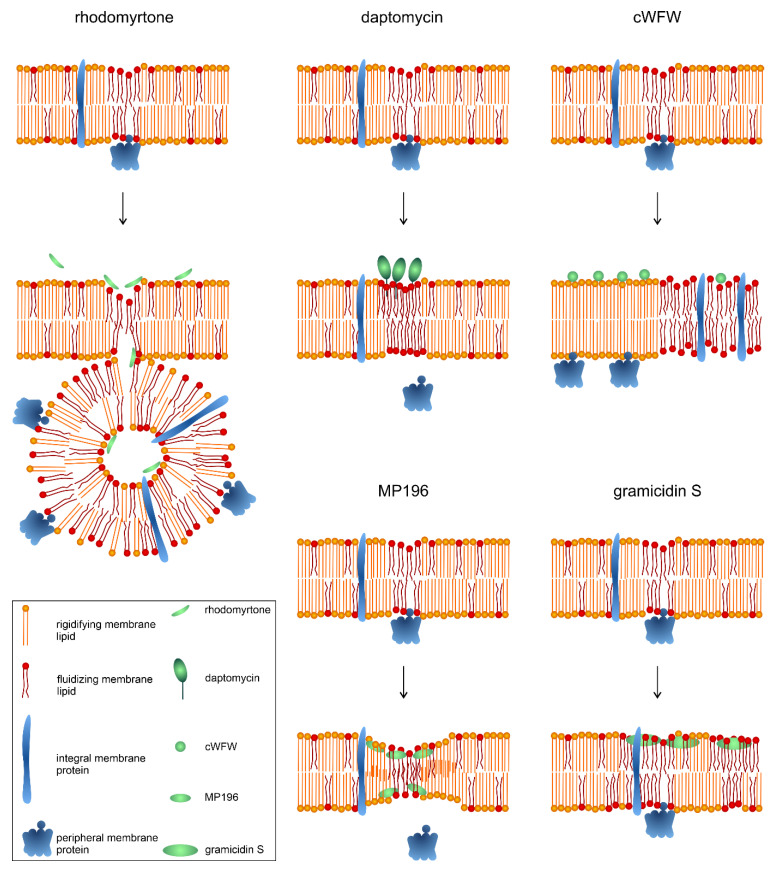
Mechanism of action of rhodomyrtone and other membrane-active compounds on bacterial cell membranes. Figure partially adapted from [78].

Figure 5 shows schematic depictions of the mechanisms of rhodomyrtone, daptomycin, cWFW, MP196, and gramicidin S. All of the compound’s membrane interactions can be described with variations of the interfacial activity model, which is characterized by the insertion of the compound at the interface of phospholipid headgroups and fatty acyl chains without spanning the whole membrane [75]. Therefore, rhodomyrtone has the most superficial membrane interaction, only transiently binding to phospholipid head groups [25], while cWFW, MP196, and gramicidin S reside at the headgroup-fatty acyl chain interface [71,73,76], and daptomycin penetrates deeper and is even able to flip to the inner membrane leaflet [77].

Another crucial commonality is the induction of membrane fluidity changes, in particular phase separation, and resulting effects on membrane protein localization (Figure 5). Thus, rhodomyrtone induces hyperfluid membrane domains that attract and trap membrane proteins in vesicles. Timelapse microscopy has shown that hyperfluid domains originate from RIFs, suggesting an affinity or increased vulnerability of RIFs to rhodomyrtone [25]. Daptomycin displays a clear affinity for RIFs due to the enrichment of PG and lipid II, both of which are bound by daptomycin [79]. Upon insertion into these domains, it clusters them together and rigidifies the formerly fluid domains into large rigid clusters. This sequesters both PG and lipid II and affects RIF-associated proteins, in particular, the lipid II synthase MurG and the phospholipid synthase PlsX, resulting in the inhibition of cell wall and membrane synthesis and concomitant cell shape defects [72,80]. cWFW causes large-scale phase separation into large membrane domains of lower fluidity, sometimes spanning the majority of the cell surface. These domains separate peripheral and integral membrane proteins, thereby disrupting membrane protein complexes like the cell wall synthesis and division machinery [73]. MP196 is thought to act closely to the originally proposed interfacial activity model, causing a funnel-like membrane constriction, allowing the slow passage of ions by jumping from hydrophilic residue to hydrophilic residue [71,75]. This deformation causes the displacement of peripheral membrane proteins into the cytosol, which has been shown for MurG, cytochrome *c*, and the cell division regulation protein MinD [71]. Gramicidin S induces large fluid membrane domains. This phase separation causes the partitioning of membrane proteins into the fluid phase, resulting in a similar inhibition of membrane function by disruption of protein complexes and accumulation in a confined space [74]. Similar to daptomycin and MP196, gramicidin S also displaced the lipid II synthase MurG into the cytosol, resulting in cell wall defects [71,74].

While the type of phase separation, the specific fate of the affected membrane proteins, and the molecular mechanisms by which these effects are achieved differ between the compounds, they all cause the impairment of a multitude of membrane-associated processes. This overarching mechanism may explain why all five compounds have been claimed to have low resistance development rates [30,70,72,73,81]. For so far unknown reasons, cell wall synthesis has been observed to be particularly sensitive to the membrane effects of daptomycin, cWFW, MP196, and gramicidin S, but not rhodomyrtone [25,71,72,73,74].

Another similarity between the compounds is their effect on the membrane potential. Thus, MP196 and gramicidin S depolarize immediately and completely, likely due to ion translocation through an interfacial activity funnel paired with respiratory chain inhibition [71,74]. Rhodomyrtone shows a slightly delayed (~2 min), incomplete depolarization due to gradual potassium release, likely through phase separation defects [25]. While a similar mechanism involving phase boundary defects has been proposed for daptomycin, it shows extremely slow (~30 min) and incomplete depolarization [72]. Depolarization by cWFW is immediate but partial and transient (recovery within ~15 min) [73]. While the timeframe and extent of depolarization vary greatly, the added effects of membrane potential dissipation and corresponding energy limitation add two more dimensions to the mechanisms of action of these compounds. Firstly, the loss of membrane binding of membrane potential-sensitive peripheral membrane proteins such as FtsA, SepF (and consequently FtsZ), MinD, and MreB [68] additionally impairs cellular processes, mainly cell division and lateral cell wall synthesis. This effect is exacerbated by the rigidification of parts of the cell membrane, a consequence of phase separation [72]. Secondly, the depletion of ATP levels additionally impairs cellular functions and hampers the induction of appropriate stress responses [25]. These effects further contribute to the mechanistic complexity of these compounds, explaining their multiple cellular effects and low resistance development rates.

Despite some shared cellular effects, the molecular mechanisms by which they are achieved differ greatly between these compounds, and rhodomyrtone’s membrane protein nanotrap mechanism truly stands out as unique.

### 5.4. Antivirulence Activities

Several studies have provided evidence that in addition to its direct antibacterial action, rhodomyrtone may also possess antivirulence properties. Thus, a proteomic study on MRSA found differential regulation of many proteins, including cell wall synthesis and cell division, matching with the current mode of action model, but also cell surface antigens and virulence factors [29]. Similar effects were observed in *S. pyogenes*. In this pathogen, proteomic profiling revealed the down-regulation of known virulence factors like glyceraldehyde-3-phosphate dehydrogenase, CAMP factor, and streptococcal pyrogenic exotoxin C [57].

Leejae et al. showed that rhodomyrtone inhibits the synthesis of staphyloxanthin, a pigment that possesses antioxidant properties and aids *S. aureus* to escape killing by reactive oxygen species and host neutrophils [82]. Indeed, rhodomyrtone-treated bacteria were more susceptible to killing by peroxide and singlet oxygen and exhibited reduced survival rates in human whole-blood [83]. It has been speculated that the inhibition of staphyloxanthin production may be a consequence of the rhodomyrtone-mediated downregulation of DnaK or sigma factor B (SigB) [29,83]. Indeed, the inhibition of SigB activity by rhodomyrtone was confirmed in exponentially growing *S. aureus* cells [35].

Rhodomyrtone was further shown to inhibit lipase and protease production in *P. acnes* [22]. Both enzymes are secreted by *P. acnes* and contribute to inflammatory acne and skin lesions [84]. Rhodomyrtone also impaired adhesion and invasion of *S. pneumoniae* to A549 human alveolar epithelial cells and increased phagocytosis by RAW264.7 macrophages by 90–99% [55].

It should be noted that none of these effects is due to a direct inhibition of a specific virulence factor or pathway. Rather, all observed effects can be attributed to the bacterial stress response to rhodomyrtone exposure. Thus, it must be considered that these mechanisms will only take effect at subinhibitory concentrations that still allow the cells to perform differential gene expression.

## 6. Rhodomyrtone Resistance

**Figure 6:**
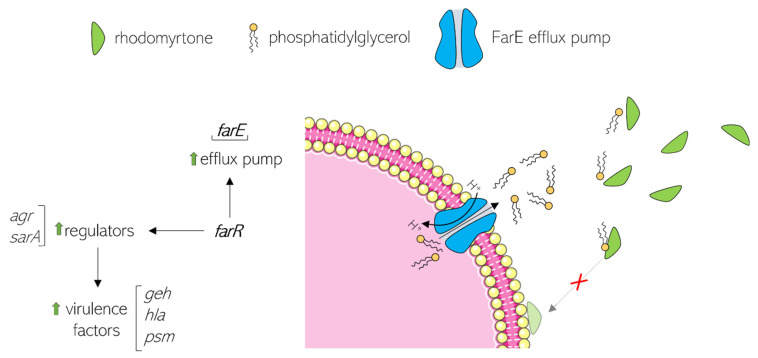
Resistance mechanism of *S. aureus* against rhodomyrtone according to results by Nguyen et al. and Huang et al. [85,87]. Green arrows indicate upregulation.

While previous attempts have failed to detect rhodomyrtone resistance [30], Nguyen et al. isolated stable, spontaneous, rhodomyrtone-resistant *S. aureus* mutants. Resistance to rhodomyrtone is based on a single point mutation in the coding region of the *farR* gene encoding the FarR regulator of fatty acid resistance, resulting in a change of Cys116 to Arg. This exchange affects FarR activity, in turn affecting other global regulators, and leads to the de-repression of *farE* (effector of fatty acid resistance) [85]. FarE is an efflux pump that confers resistance to the antimicrobial fatty acids linoleic and arachidonic acid [86]. Its overexpression in the *farR* mutant enhanced the excretion of lipids [85]. Qualitative and quantitative lipidomic profiling revealed that the *farR* mutant released 10 times more phospholipid into the surrounding medium than its parent strain [87]. It has been hypothesized that the increased excretion of lipids neutralizes rhodomyrtone activity since rhodomyrtone binds to phospholipid head groups [25]. In line with this model, the antimicrobial activity of rhodomyrtone is diminished in the presence of the fatty acids pentadecylic acid, palmitic acid, and stearic acid [58]. The current model of this resistance mechanism is illustrated in Figure 6.

Based on the current model, cross-resistance with other membrane-binding antibiotics would be expected, in particular with daptomycin, for which a similar resistance mechanism has been described [88]. However, this was not the case, and the *farR* mutant seemed to specifically confer resistance to rhodomyrtone [87], indicating that there are additional factors involved in this mechanism of resistance that so far escape our notice. It should be noted that some rhodomyrtone derivatives synthesized by Wenninger et al. were indifferent to the *farR* mutation and retained full activity against the mutated strain [50]. However, it has not yet been assessed whether these derivatives retain the same mechanism of action as rhodomyrtone or not.

Another consequence of altered FarR activity is the upregulation of the *agr* and *sarA* genes, which in turn increase the expression of the virulence genes *geh* (lipase), *hla* (alpha-hemolysin), and *psm* (phenol soluble modulin) (Figure 6), resulting in significantly higher cytotoxicity and pathogenicity of the *farR* mutant compared to its parent strain in a mouse model [85]. This is an unfavorable finding as exposure to rhodomyrtone may not only result in resistance but concomitantly increase the virulence and pathogenicity of resistant strains. Whether such an effect is specific to *S. aureus* or may also occur in other species remains to be examined.

## 7. Toxicity

The enormous diversity of reported activities of rhodomyrtone carries an inherent risk of toxicity as it suggests that the compound can interact with a number of structures instead of one specific target. However, most studies indicate that rhodomyrtone possesses low toxicity, reinforcing its route to application in the clinic.

In a toxicity study aimed at topical use, the toxicity of *R. tomentosa* extract and purified rhodomyrtone was determined against human fibroblasts. IC_50_ values of 476 and >200 mg/mL, respectively, left therapeutic windows of 15 and 400-fold based on MIC_90_ values, indicating very low cytotoxicity of rhodomyrtone [20]. Also, rhodomyrtone did not cause hemolysis up to 256 µg/mL [89]. However, one study showed that rhodomyrtone is cytotoxic for several eukaryotic cell types and can induce eryptosis accompanied by erythrocyte shrinkage, cell membrane blebbing, and membrane scrambling with phosphatidylserine translocation to the erythrocyte surface [58]. The reasons behind these discrepancies are yet to be determined.

Despite these mixed results, acute toxicity tests in diverse topical and systemic animal models have shown favorable results. Thus, rhodomyrtone did not cause skin irritation in rabbits [90]. Rhodomyrtone did also not cause any visible toxic effects upon injection into *Galleria mellonella* (greater wax moth larvae) at 100 mg/kg of body weight for up to four days [89]. Similarly, no acute toxicity was observed upon injection into either the tail vein or yolk circulation valley of zebrafish embryos for up to three days [25,30]. Even in mammalian models, no toxic effects were found. Thus, no systemic toxicity was observed in mice that received a high oral dose of 5000 mg/kg body for up to 14 days [89]. Another mouse study aimed at evaluating the antidepressant effects of rhodomyrtone used daily intraperitoneal doses of 15 mg/kg for three weeks and did not find adverse effects [91]. A recent rat study aimed to assess of the pharmacokinetics of rhodomyrtone, orally administered single doses of 50 and 100 mg/kg of body weight. No adverse effects were observed. Notably, blood plasma concentrations of rhodomyrtone were above the MIC values for most Gram-positive pathogens [92].

It must also be noted that *R. tomentosa* leaf concoctions have been used in traditional Asian medicine for ages, suggesting that rhodomyrtone is safe for oral consumption by humans, at least at doses that correspond to the natural remedies [13,14,15].

## 8. Potential Therapeutic Effects in Mammalian Cells

**Figure 7:**
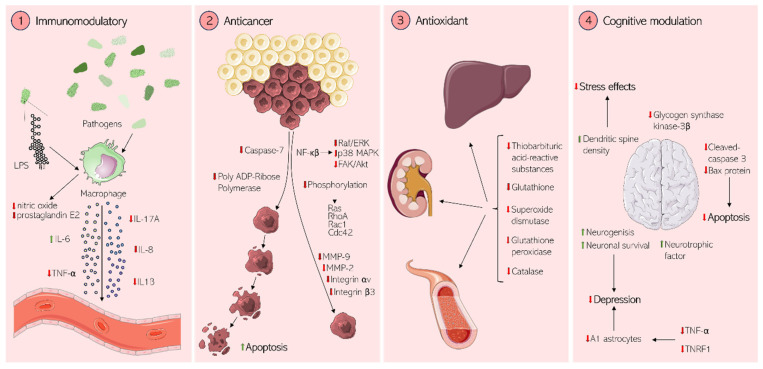
Effects of rhodomyrtone on mammalian cells. (**1**) Immunomodulatory, (**2**) anticancer, (**3**) antioxidant, and (**4**) cognitive effects of rhodomyrtone. Red arrows indicate down-regulation.

In addition to antibacterial applications, several other medical uses of rhodomyrtone have been proposed based on its activities in mammalian systems (Figure 7). These comprise immunomodulatory, anticancer, cognitive, and antioxidant properties.

### 8.1. Immunomodulation

Immunomodulation is a highly sought-after property of new-generation anti-infective drugs since they, in addition to reducing undesired inflammatory activity, recruit the body’s innate immune system to fight off infections. The immunomodulatory activities of rhodomyrtone have been explored and characterized in several studies (Figure 7). Jeong et al. examined the in vitro and in vivo anti-inflammatory effects of *R. tomentosa* extract in the murine macrophage cell line RAW264.7, murine primary peritoneal macrophages, and mouse gastritis and acute ulcerative colitis models. The authors observed that *R. tomentosa* extract inhibited the production of nitric oxide and prostaglandin E2 in both lipopolysaccharide (LPS)-activated RAW264.7 cells and peritoneal macrophages in a dose-dependent manner. Further, suppression of both nuclear factor κB (NF-κB) and activator protein-1 (AP-1) pathway activation was observed in both cell and mouse models [93]. Anti-inflammatory effects were also observed in rainbow trout head kidney cells. Na-Phatthalung et al. reported expression changes of both pro-inflammatory (interleukin 1β (IL-1β), IL-8, tumor necrosis factor α (TNFα)) and anti-inflammatory cytokines (IL-10 and transforming growth factor β (TGF-β)) upon exposure to either *R. tomentosa* leaf extracts or rhodomyrtone. A down-regulation of inflammatory responses (IL-1β, IL-8, TNF-α, iNOS, saa, hepcidin, and gpx1) upon co-exposure to rhodomyrtone and LPS further corroborated its anti-inflammatory effects [94].

It was further shown that rhodomyrtone had both pro- and anti-inflammatory effects on THP-1 cells (human leukemia monocyte cell line) that were stimulated with heat-inactivated MRSA. Thus, rhodomyrtone increased the expression of the pro-inflammatory mediators IL-6 and the nitric oxide synthase iNOS and decreased the expression of TNF-α. This modulation of pro- and anti-inflammatory cytokine responses promoted the phagocytosis of MRSA by THP-1 monocytes, suggesting that immunomodulation by rhodomyrtone may help clear infection in the host [95].

Rhodomyrtone has been suggested as a possible treatment for psoriasis, an autoimmune skin condition caused by TNF-α and IL-17A-induced epidermal hyperproliferation and inflammatory responses. Its pathogenesis is characterized by the proliferation of the basal epidermal layer and abnormal keratinocyte differentiation. Using HaCaT keratinocytes (an immortalized human keratinocyte cell line), Chorachoo et al. showed that rhodomyrtone exhibits anti-proliferative effects on and induced apoptosis of keratinocytes [96]. A follow-up study demonstrated that rhodomyrtone significantly lowered the expression and secretion of inflammatory proteins in human skin organ models stimulated with TNF-α and IL-17A, probably by modulating mitogen-activated protein kinase (MAPK) and NF-κB signaling pathways. Further, rhodomyrtone reversed imiquimod-induced skin hyperplasia and epidermal thickening in mice with imiquimod-induced skin inflammation, suggesting that it has the potential to be developed into an effective treatment for psoriasis [90].

### 8.2. Anticancer Activity

Rhodomyrtone has also been investigated with respect to anticancer activities. Tayeh et al. reported that rhodomyrtone inhibited the proliferation of human epidermoid carcinoma A431 (skin cancer) cells in a dose-dependent manner. Using a wound healing assay, the authors showed that rhodomyrtone reduced the migration of A431 cells, caused cell arrest in the G1 phase, and induced apoptosis, apparent through membrane blebbing, chromatin condensation, and cell shrinkage. Apoptosis was induced by the cleavage of caspase-7 (CASP7) and poly (ADP-ribose) polymerase (PARP) [97]. Additionally, rhodomyrtone inhibited A431 metastasis by inhibiting Raf/extracellular signal-regulated kinase (Raf/ERK), p38 MAPK, and focal adhesion kinase/Akt (FAK/Akt) signaling pathways via NF-κB activities [98]. These results suggested that rhodomyrtone could possibly be used to treat skin cancer.

Tayeh and Watanapokasin further studied the antimetastatic activities of rhodomyrtone in human chondrosarcoma SW1353 (bone cancer) cells and observed a significant, dose-dependent reduction of SW1353 viability, migration, invasion, and adhesion. This was attributed to a suppression of integrin αvβ3/FAK/Akt/small Rho GTPase pathways as well as the downregulation of matrix metalloproteinases 2 and 9 (MMP-2/9) via extracellular signal regulation kinase (ERK) and c-Jun N-terminal kinase (JNK) signal inhibition, as evidenced by protein expression analysis [99].

### 8.3. Antioxidant Activity

In addition to its use against infections, *R. tomentosa* is also traditionally used for skin beauty treatments, e.g., for skin whitening and anti-aging [100]. These activities have been ascribed to antioxidant activities. In vitro studies could indeed confirm the antioxidant properties of rhodomyrtone, evidenced by reduced lipid peroxidation, strong ferric-reducing properties, and ferrous ion chelation. In vivo studies in Swiss Albino mice treated with carbon tetrachloride (CCl_4_) revealed that rhodomyrtone was capable of reversing the effects of CCl_4_ treatment, including (partial) restoration of the levels of thiobarbituric acid-reactive substances (TBARS), glutathione (GSH) and the antioxidant enzymes superoxide dismutase (SOD), catalase (CAT), and glutathione peroxidase (GPx) in the blood, liver, and kidneys of the animals. These observations suggest that rhodomyrtone has direct antioxidant properties and counteracts oxidative stress in mice [100].

### 8.4. Cognitive and Neuronal Effects

Rhodomyrtone may also display beneficial cognitive effects, even though this area has been very little explored so far. Yet, a study by Chai et al. has suggested that rhodomyrtone can prevent depression-like behaviors and impairment of spatial memory in mice exposed to chronic, unpredictable, mild stress. These effects were attributed to a reversal of dendritic spine density defects, inhibition of the increase in glycogen synthase kinase-3β activity, reversal of a decrease in brain-derived neurotrophic factor and postsynaptic density protein 95, and reversal of elevated expression of apoptosis-associated protein Bax and cleaved-caspase 3, all of which are associated with depressive disorders. This pilot study has provided evidence for an antidepressant activity of rhodomyrtone that involves the promotion of neurogenesis and neuronal survival in the hippocampus and paved the way for further studies into these properties [91]. A recent study correlated the antidepressant effects of rhodomyrtone to the repression of tumor necrosis factor receptor 1 (TNFR1) and TNF-α. TNF- α is an activator of A1 astrocytes, which have been implicated in depression, offering a possible mechanism for the antidepressant activity of rhodomyrtone [101].

## 9. Conclusions

Rhodomyrtone is a multifunctional molecule with a range of pharmaceutic applications. While its antibacterial activities and mechanisms are by far the most well-characterized, the compound possesses further activities that have been less thoroughly explored, including antiplasmodial, immunomodulatory, anticancer, antioxidant, and even cognitive effects. Certainly, these additional activities will be subject to further research to unlock the full potential of this exceptional drug. However, even in terms of antibacterial applications, open questions remain. Thus, the molecular mechanism by which rhodomyrtone induces hyperfluid membrane domains and subsequent vesiculation is not well understood. Due to the uniqueness of this mechanism, a better understanding of the molecular determinants of this activity would be greatly beneficial, in particular for understanding the structure-activity relationship, which would enable more efficient design of promising derivatives with modulated activities. Careful research must be conducted with respect to the described resistance mechanism by mutation of *farR* in *S. aureus*. The observation that there is no cross-resistance with other membrane-active compounds suggests that there is more to this mechanism than meets the eye. It is also surprising that this phenotype can be achieved by a simple point mutation and is stable, considering that other studies have failed to generate stable rhodomyrtone-resistant *S. aureus* mutants, even after extensive passaging. Further, the concomitant increase in pathogenicity is concerning, and its implications in clinical scenarios must be carefully assessed. Finally, the discrepancy between different cytotoxicity studies must be addressed. While all published animal studies have shown no reason for concern and *R. tomentosa* extract has been safely consumed by humans for a long time, it is surprising that significant toxicity has been observed for different eukaryotic cell types. This points to a so far hidden activity or property of rhodomyrtone. It could be speculated that the enantiomer ratio, purity, or stability of the compound could be factors underlying such conflicting observations. In order to thoroughly assess the safety of rhodomyrtone as a manufactured drug, these factors must be carefully assessed. Taken together, rhodomyrtone is an exceptional molecule with great promise but also potential bottlenecks that must be addressed prior to its advancement in the clinical pipeline. It is a good example of the potency of plant-derived antimicrobial agents and will hopefully inspire further research into the plant kingdom as a source of new lead compounds to combat AMR.
